# The Role of the Two-Component System PhoP/PhoQ in Intrinsic Resistance of *Yersinia enterocolitica* to Polymyxin

**DOI:** 10.3389/fmicb.2022.758571

**Published:** 2022-02-10

**Authors:** Haoran Guo, Tong Zhao, Can Huang, Jingyu Chen

**Affiliations:** Beijing Laboratory for Food Quality and Safety, College of Food Science and Nutritional Engineering, China Agricultural University, Beijing, China

**Keywords:** *Yersinia enterocolitica*, PhoP/PhoQ, LPS modification, polymyxin, intrinsic resistance

## Abstract

Polymyxin is the “last resort” of antibiotics. The self-induced resistance to polymyxin in Gram-negative bacteria could be mediated by lipopolysaccharide (LPS) modification, which is regulated by the two-component system, PhoP/PhoQ. *Yersinia enterocolitica* is a common foodborne pathogen. However, PhoP/PhoQ has not been thoroughly studied in *Y. enterocolitica*. In this study, the functions of PhoP/PhoQ in *Y. enterocolitica* intrinsic resistance were investigated. The resistance of *Y. enterocolitica* was found to decrease with the deletion of PhoP/PhoQ. Further, PhoP/PhoQ was found to play an important role in maintaining membrane permeability, intercellular metabolism, and reducing membrane depolarization. Based on subsequent studies, the binding ability of polymyxin to *Y. enterocolitica* was decreased by the modification of LPS with structures, such as L-Ara4N and palmitate. Analysis of the gene transcription levels revealed that the LPS modification genes, *pagP* and *arn* operon, were downregulated with the deletion of PhoP/PhoQ in *Y. enterocolitica* during exposure to polymyxin. In addition, *pmrA*, *pmrB*, and *eptA* were downregulated in the mutants compared with the wild-type strain. Such findings demonstrate that PhoP/PhoQ contributes to the intrinsic resistance of *Y. enterocolitica* toward polymyxins. LPS modification with L-Ara4N or palmitate is mainly responsible for the resistance of *Y. enterocolitica* to polymyxins. The transcription of genes related to LPS modification and PmrA/PmrB can be both affected by PhoP/PhoQ in *Y. enterocolitica*. This study adds to current knowledge regarding the role of PhoP/PhoQ in intrinsic resistance of *Y. enterocolitica* to polymyxin.

## Introduction

*Yersinia enterocolitica* is a common foodborne pathogen that causes a broad range of gastrointestinal syndromes in humans (yersiniosis; Shoaib et al., 2019). *Yersinia enterocolitica* is widely distributed in various environments and food production (Shoaib et al., 2019). To infect humans, *Y. enterocolitica* must survive in the host environment by escaping cationic antibiotic peptides (AMPs), which mediate parts of the innate immune system against infections (Rahnamaeian, 2011). In bacteria, two-component systems (TCSs) are employed to sense and respond to various environmental stresses (West and Stock, 2001). Typical TCSs consist of a membrane-embedded sensor kinase and a cytosolic response regulator. These systems can sense extracellular signals and perform cascade phosphorylation in response. The activated response regulator then regulates the expression of specific genes directly or indirectly, thereby initiating cellular responses to environmental changes (Hoch, 2000; West and Stock, 2001).

**Figure 1:**
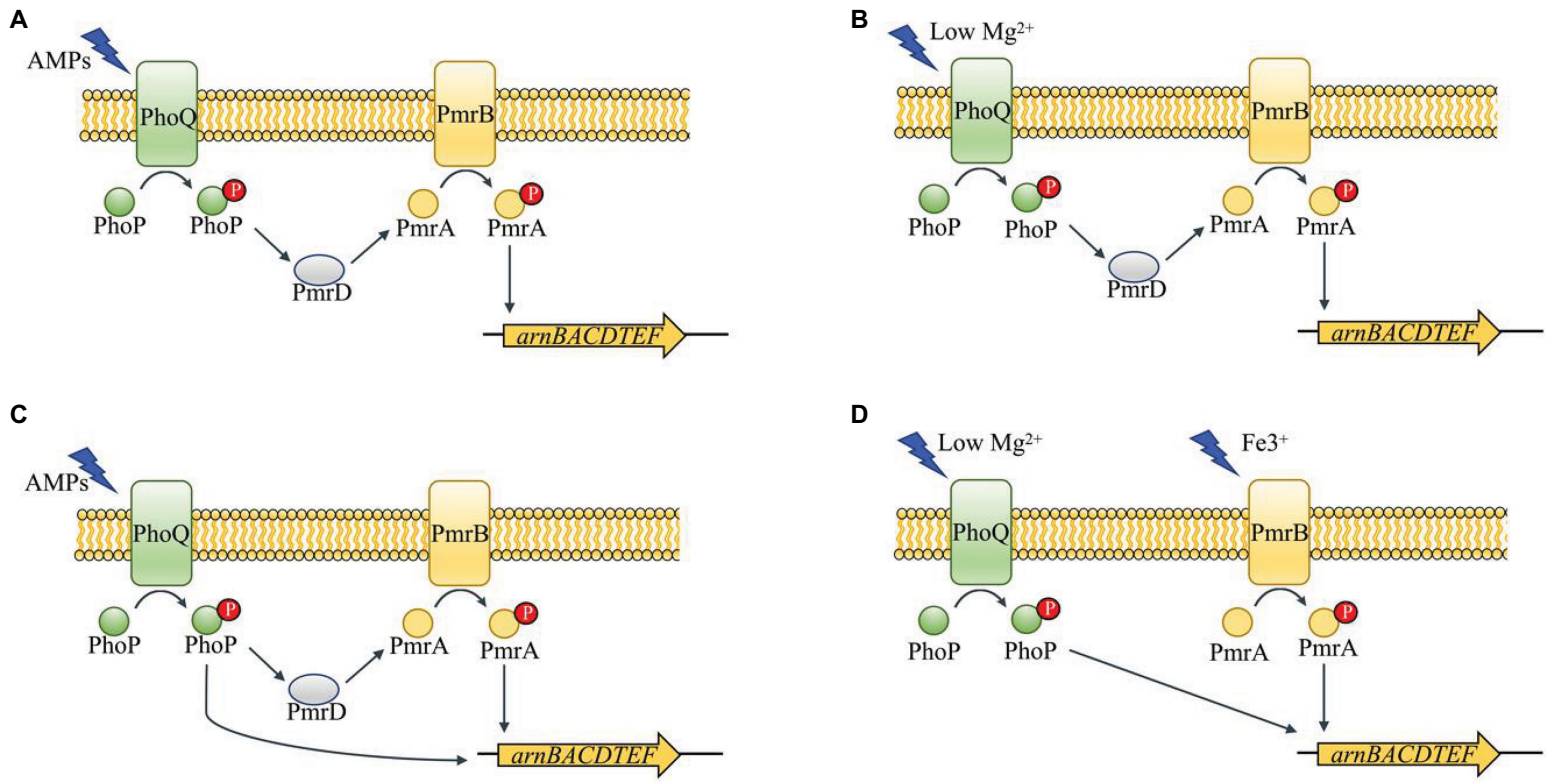
Schematic of PhoP/PhoQ and PmrA/PmrB regulation network in different genera of Enterobacteriaceae (Winfield et al., 2005; Rubin et al., 2015; Hong et al., 2018; Chen et al., 2021). PmrD functions as a connector between PhoP/PhoQ and PmrA/PmrB in *Salmonella enterica* **(A)**. The connector function of PmrD is activated under low Mg^2+^ in *Escherichia coli* **(B)**. PhoP can regulate *arn* operon directly and indirectly in *Klebsiella pneumoniae* **(C)**. PhoP/PhoQ and PmrA/PmrB perform a cross-regulation of *arn* operon under different environments in *Yersinia pestis* **(D)**.

PhoP/PhoQ is a prototypical TCS present in many bacteria. PhoQ, the dimeric sensor kinase of this system, contains cytoplasmic, transmembrane, and periplasmic domains (Véscovi et al., 1997; Bader et al., 2005; Prost et al., 2007). The patch of acidic residues close to the membrane surface in the periplasmic sensing domain of PhoQ is crucial for sensing cationic AMPs (Bader et al., 2005). Divalent cation concentrations, low pH conditions, and high osmolarity have been reported to activate PhoQ (Véscovi et al., 1997; Bader et al., 2005; Prost et al., 2007). And PhoP is a phosphorylated response regulator which is controlled by activated PhoQ (Simpson and Trent, 2019). PhoP directly regulates a set of genes that vary among different bacteria (Winfield et al., 2005; Hong et al., 2018; Chen et al., 2021). However, some Gram-negative bacteria have conserved ancestral genes, such as *mgtA* in *Escherichia coli* acting as magnesium transporter and *mgrB* as the direct negative regulator of PhoP/PhoQ (Minagawa et al., 2003; Zwir et al., 2012). The protein, PmrD, which is also directly regulated by PhoP, could combine PhoP/PhoQ and PmrA/PmrB. Thus, PhoP/PhoQ is indirectly involved in PmrA/PmrB regulation of genes (Figures 1A,B; Rubin et al., 2015; Hong et al., 2018).

Polymyxins are AMPs that target Gram-negative outer membranes (Mitchell and Silhavy, 2019). Lipid A of Gram-negative bacterial outer membrane is the target binding site for AMPs, which mediates the interaction with negatively charged LPS. A hydrophobic domain is known to insert itself into membranes, forming pores, which results in cell lysis and death (Brogden, 2005). The resistance of Gram-negative bacteria to polymyxins primarily relies on chemical modifications of the lipopolysaccharide (LPS) structure and leads to major changes in the physicochemical properties of the outer membrane (Trimble et al., 2016). In most resistant strains, LPS modified with 4-amino-4-deoxy-L-arabinose (L-Ara4N), phosphoethanolamine (pEtN), as well as other modification substituents, showed polymyxin resistance (Santos et al., 2017). Expression of most of the genes in the LPS modification pathway is controlled by several TCSs, including PhoP/PhoQ and PmrA/PmrB (Rubin et al., 2015; Trimble et al., 2016; Duperthuy, 2020).

Although PhoP/PhoQ has been best studied in *Salmonella* typhimurium, it has not been thoroughly studied in *Y. enterocolitica*. Further, some proteins such as PmrD in PhoP/PhoQ regulatory pathway in *Salmonella* have not been found in *Y. enterocolitica*. Therefore, whether the resistance of *Y. enterocolitica* to polymyxins could be affected by the deletion of PhoP/PhoQ needs to be determined. Further, the role of PhoP/PhoQ in the intrinsic resistance mechanism of *Y. enterocolitica* to AMP is unclear. In this study, the role of PhoP/PhoQ in the polymyxin resistance of *Y. enterocolitica* as well as the growth, membrane damage, and intracellular metabolism of *Y. enterocolitica* was identified. The difference in the ability of polymyxins to bind to resistant or sensitive membranes regulated by PhoP/PhoQ was assessed. In addition, *Y. enterocolitica* lipid A modifications implicated in polymyxin resistance were characterized. By analyzing gene expression, PhoP/PhoQ was found to affect polymyxin resistance by regulating various genes related to LPS modification. These findings reveal some of the roles of PhoP/PhoQ in *Y. enterocolitica* polymyxin resistance.

## Materials and Methods

### Bacterial Strains, Plasmids, and Culture Conditions

The bacterial strains and plasmids used in this study are listed in Table 1. *Escherichia coli* S17-λpir was used as the host bacteria in plasmid construction and was cultured at 37°C in lysogeny broth (LB) containing 5 g/L yeast extract, 10 g/L tryptone, and 5 g/L NaCl. *Yersinia enterocolitica* ATCC23715 (biotype 1 B and serotype O:8) was used as the parent strain for the construction of *Y. enterocolitica* mutants, which were cultured at 26°C in LB broth and LBNS (LB without salts). Ampicillin (100 μg/ml), chloramphenicol (16 μg/ml), cefsulodin (15 μg/ml), irgasan (4 μg/ml), and novobiocin (2.5 μg/ml) were added as required.

**Table 1:** Strains and plasmids used in this study.

Strains and plasmids	Relevant characteristics	Sources
*Yersinia enterocolitica*		
ATCC23715	WT, serotype O:8, Biotype 1B, pYV^−^	Lab stock
Δ*phoP*	Δ*phoP*	This study
Δ*phoP*-*phoP*	Δ*phoP*, P_BAD_*phoP*; Amp^r^	This study
Δ*phoQ*	Δ*phoQ*	This study
Δ*phoQ-phoQ*	Δ*phoQ*, P_BAD_*phoQ*; Amp^r^	This study
*Escherichia coli*		
S17-1 λpir	*recA1*, *thi*, *pro*, *hsdR*-M^+^, RP4:2-Tc:Mu^−^Kan:Tn7, λpir	Lab stock
Plasmids		
pDS132	Conditional replication vector; R6K origin, mobRK4 transfer origin, sucrose-inducible-*sacB*; Cm^r^	Lab stock
pDS132-Δ*phoP*	Upstream and downstream *phoP* fragments were cloned into pDS132; Cm^r^	This study
pDS132-Δ*phoQ*	Upstream and downstream *phoQ* fragments were cloned into pDS132; Cm^r^	This study
pBAD24	*AraC*, promoter P_BAD_; Amp^r^	Lab stock
pBAD24-*phoP*	*AraC*, P_BAD_*phoP*; Amp^r^	This study
pBAD24-*phoQ*	*AraC*, P_BAD_*phoQ*; Amp^r^	This study

*Amp^r^, ampicillin resistance and Cm^r^, chloramphenicol resistance*.

### Construction of Plasmids and Strains

To construct pDS132-Δ*phoP*, fragments upstream and downstream of the *phoP* gene were amplified from the *Y. enterocolitica* genome using the primers, *phoP-up-F/phoP-up-R* and *phoP-down-F/phoP-down-R*. The upstream and downstream fragments were fused and amplified by fusion PCR with primers *phoP-up-F/phoP-down-R*. The resultant long fragment was then digested with *Sac*I and *Sal*I and ligated into pDS132 digested with the same enzymes to yield pDS132-Δ*phoP*. The same approach was used to construct pDS132-Δ*phoQ* with the corresponding primers. To construct the *phoP* knockout strain, the suicide plasmid, pDS132-Δ*phoP*, was introduced into *E. coli* S17-1 λpir by electroporation and then mobilized into *Y. enterocolitica* by conjugation. The strategy used for gene deletion in the *Y. enterocolitica* chromosome was based on the two-step homologous recombination with plasmid pDS132 containing the *sacB* counter-selectable marker and a chloramphenicol resistant marker, as described previously (Schäfer et al., 1994). Similarly, the plasmid pDS132-Δ*phoQ* was used to construct the Δ*phoQ* mutants.

To construct pBAD24-*phoP*, the *phoP* fragment was amplified from the *Y. enterocolitica* genome using primers *p-phoP-F*/*p-phoP-R*, digested with *Xbal*I and *Hin*dIII, and inserted into pBAD24 digested with the same enzymes. The resultant pBAD24-*phoP* plasmid contained the *phoP* gene, which was controlled by the *araBAD* promoter (P*BADphoP*). The plasmid, pBAD24-*phoP*, was used to transform the Δ*phoP* mutant by electroporation to construct the Δ*phoP-phoP* complemented strain. The same method was also used to construct Δ*phoQ*-*phoQ* complemented strains. All primers used in this study are listed in Supplementary Table 1. Ampicillin (100 μg/ml) and L-arabinose (0.6 g/L) were added to maintain plasmid and induce the expression of *phoP* or *phoQ*, respectively.

### Growth Curves Measurement

Growth curves of the strains were determined by measuring the OD_600_ at 60 min intervals over a period of 24 h. Overnight cultures of bacterial strains were inoculated into LB medium supplemented 0.5 μg/ml PMB or PME (at 1:50 dilution) and incubated at 26°C with shaking at 180 rpm. Growth curves under no PMB or PME were also measured for the control group. L-arabinose (0.6 g/L) was added to induce the expression of *phoP* or *phoQ* of complemented strains. The experiments were performed with three biological replicates.

### Minimal Inhibitory Concentration and Minimal Bactericidal Concentration

The minimal inhibitory concentration (MIC) of PMB and PME were assessed according to the CLSI guidelines with some modifications (CLSI, 2017). Briefly, overnight cultures of WT, *phoP*, and *phoQ* mutants were diluted in cation-adjusted Mueller–Hinton broth (CaMHB) with approximately10^6^ colony forming units (CFU)/ml in tubes. Then, the suspended bacteria were exposed to PMB or PME at 0.5, 1.0, 2.0, 4.0, 8.0, and 16.0 μg/ml. All tubes were incubated for 18–24 h at 26°C with shaking. The MIC was determined as the minimal concentration at which turbidity was not visible in comparison with the blank and minimal bactericidal concentration (MBC) was the minimal concentration which had no growth. And it was performed with three biological replicates.

### Laser Scanning Confocal Microscope

The overnight cultures were inoculated to LBNS broth and grown at 26°C and 180 rpm to mid-log phase. The bacterial cells were collected and washed with PBS buffer for three times and then resuspended at a final optical density of OD_600_ = 1.0. The equal volume of bacterial suspension was mixed with PMB or PME solution (10 μg/ml) and incubated at 26°C for 2 h. Then, the solutions were centrifuged at 5,000 × *g* for 5 min and resuspended at the same volume with PBS. And cell viability after PMB or PME treatment was assessed using fluorescently labeled propidium iodide (PI) and 4′,6-diamidino-2-phenylindole (DAPI) and analyzed with a laser scanning confocal microscope (LSCM). DAPI is a blue, fluorescent stain that labels both live and dead cells while PI is a red fluorescent nucleic acid stain that only penetrates cells with damaged cell membranes (dead cells). All images were captured by LSCM (Leica TCS SP8, Mannheim, Germany) and quantified using ImageJ.

### Cell Membrane Permeabilization and Potential

N-phenyl-1-naphthylamine (NPN) uptake assays were performed to evaluate the outer membrane permeability of *Y. enterocolitica* strains, as previously described (Lee and Je, 2013). Briefly, cells were harvested in the log phase of growth and centrifuged at 5,000 rpm for 10 min at 4°C. Thereafter, the cells were washed three times with PBS and resuspended to an OD_600_ of 0.5. An aliquot of bacterial suspension and polymyxin solutions were fully mixed with NPN at a final concentration of 10 μM. The fluorescence value was then measured immediately using a spectrofluorometer FS5 (Edinburgh Instruments, United Kingdom). Excitation and emission wavelengths for NPN were set at 350 and 420 nm, respectively, with a slit width of 0.5 nm. The inner membrane permeability was tested with O-nitrophenyl-*β*-D-galactoside (ONPG) according to the method previously mentioned (Lee and Je, 2013). Logarithmic-phase bacteria were washed with PBS and resuspended to OD_600_ at 0.5. Then, 100 μl of bacterial suspension was added to 100 μl polymyxin solutions and 10 μl ONPG (30 mM) in each well. And the absorbance at 410 nm was used to evaluate o-nitrophenol over time using a spectrophotometer.

Overnight cultures were inoculated to LBNS and grown at 180 rpm, 26°C to mid-log phase. And bacteria were washed with 20 mM HEPES buffer for three times at 4°C and adjust to OD_600_ = 0.5. Then, bacteria were exposed under 0.5 μg/ml PMB or PME for 2 h at 26°C. Changes in membrane polarity were detected using the bis-oxonol dye, DiBAC_4_(3) (AAT Bioquest, Inc., CA, United States). The assay was performed according to a previously described method with some modifications (Clementi et al., 2014).

### Measurement of Intracellular Adenosine Triphosphate Concentration

Overnight cultures were inoculated to LBNS and grown at 180 rpm, 26°C to mid-log phase. Then, bacteria were washed with 20 mM HEPES buffer for three times at 4°C and adjust to OD_600_ = 0.5. Then, equal volume of bacteria suspension and 1 μg/ml PMB or PME solutions were incubate for 2 h at 26°C. The adenosine triphosphate (ATP) concentration of *Y. enterocolitica* after PMB or PME treatment was assayed using an ATP assay kit (Beyotime Biotechnology, Jiangsu, China). ATP remain rate was used which was calculated by dividing the ATP content of *Y. enterocolitica* treated with PMB or PME by the corresponding ATP content without PMB or PME treated.

### Dansyl-Polymyxin Binding Experiments

Dansyl-PMB and dansyl-PME were prepared according to the modified method of Schindler and Teuber (Schindler and Teuber, 1975). The synthesized Dansyl-PMB and Dansyl-PME were dissolved in 3 ml of 1× PBS buffer (pH 7.0), purified using a Sephadex G-25 column, and quantitated using the dinitrophenylation assay (Bader and Teuber, 1973). The concentrations of dansyl-PMB and dansyl-PME were 0.519 and 0.409 mg/ml, respectively, relative to a triplicate standard curve derived from a 1 mg/ml stock solution of PMB and PME.

The fluorescence of dansyl-polymyxin bound to bacteria was measured using a spectrofluorometer FS5 (Edinburgh Instruments, United Kingdom), set at an excitation wavelength of 330 nm and an emission wavelength of 565 nm. To quantify Dansyl-PMB and Dansyl-PME binding, previous methods were used as reference, with some modifications (Cullen et al., 2011; Akhoundsadegh et al., 2019). All strains were grown to an OD_600_ of 0.8 to 1.0, and the cells were washed three times with PBS. Thereafter, the same volume of Dansyl-PMB or Dansyl-PME was applied to washed cells and incubated for 2 min. Following incubation, the cells were washed with PBS and resuspended in PBS. The fluorescence was determined by spectrofluorometer FS5 and A_600_ was determined using a microplate reader (Thermo Scientific, Waltham, MA, United States). Each experiment was repeated in triplicate, and data are reported as the ratio of fluorescence intensity to A_600_.

### Lipid A Extraction and Analysis by MALDI-TOF

*Yersinia enterocolitica* cultures (200 ml) were grown to an A_600_ of 0.5 and then exposed to 1.0 μg/ml PMB or PME for 3 h. The cells were then harvested by centrifugation. Lipid A was purified from bacteria as described previously (Hankins et al., 2013; Henderson et al., 2013) and stored frozen at −20°C. The lipid A species were analyzed using a Bruker Atuoflex III MALDI-TOF mass spectrometer (Bruker Daltonics, Germany) according to a previous method (Hankins et al., 2013; Henderson et al., 2013; Han et al., 2018b).

### RNA Extraction and Real-Time Quantitative PCR

Overnight cultures were inoculated to LBNS and grown at 180 rpm, 26°C for 3 h, and then exposed to 1.0 μg/ml PMB or PME for 3 h until OD_600_ was approximately at 0.5. Total RNA was extracted from *Y. enterocolitica* strains using the TransZol Up Plus RNA Kit (TransGen, Beijing, China). The extracted RNA was then tested for concentration and quality using a Nanodrop 2000c (Thermo Scientific, Waltham, United States). Real-time quantitative PCR (RT-qPCR) was conducted using SYBR Green and specific primers (Supplementary Table 2) in a Light Cycler 480 II (Roche, Basel, Switzerland). Reactions were performed in triplicate. Relative transcription of the target genes was analyzed by the 2^–ΔΔCt^ method described previously, and the 16S rRNA gene was used as a reference for normalization (Meng et al., 2020).

### Statistical Analysis

Statistical analysis was performed using GraphPad Prism 8 (GraphPad Software, San Diego, CA, United States). One-way ANOVA analysis was performed, and the levels of significance are indicated in the figure legends.

## Results

### Deletion of PhoP/PhoQ Decreases the Resistance of *Yersinia enterocolitica* to Polymyxins

**Figure 2:**
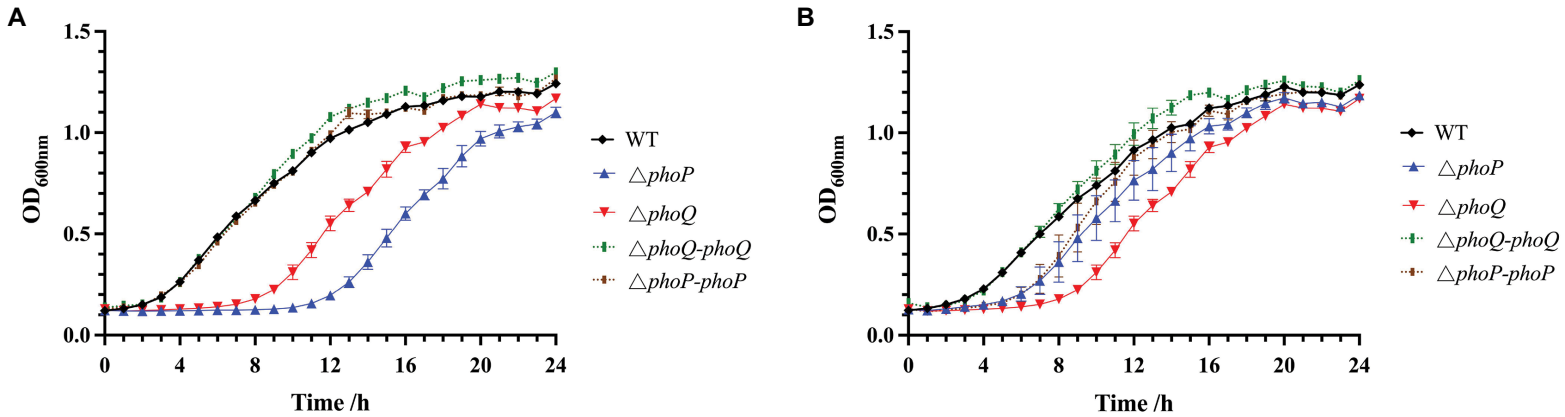
Effect of PhoP/PhoQ on the growth of *Y. enterocolitica* under PMB or PME. Growth curves of wild type, mutants and complemented strains under 0.5 μg/ml PMB **(A)**. Growth curves of wild type, mutants and complemented strains under 0.5 μg/ml PME **(B)**.

Studies have shown that the PhoP/PhoQ system can respond to the presence of antimicrobial peptides and increase resistance (Shprung et al., 2012; Lin et al., 2018). To explore the role of the PhoP/PhoQ system in *Y. enterocolitica* exposed to polymyxins, MIC and MBC of wild-type and mutants were evaluated (Table 2). MIC to PMB and PME of wild type were both 2 μg/ml, while *phoP* or *phoQ* mutants was 1 μg/ml. And MBC were also decreased when PhoP/PhoQ was deleted in *Y. enterocolitica*. Then, the growth curves of wild-type, mutants, and complemented strains were also measured. The growth of *Y. enterocolitica* was not affected by the deletion of PhoP/PhoQ (Supplementary Figure 1). However, the lag phase of the growth curves was prolonged in *phoP* and *phoQ* mutants compared to the wild-type and complemented strains under 0.5 μg/ml PMB or PME (Figure 2). Both wild-type and mutant strains were stained with PI and DAPI after treatment with 5 μg/ml of PMB or PME and observed using LCSM (Supplementary Figure 2). The live-to-dead bacterial ratio was quantitatively analyzed, and the ratios of the mutants were lower than those of the wild type. Combined with the above evidence, the deletion of the PhoP/PhoQ system was found to decrease the resistance of *Y. enterocolitica* to PMB and PME and affected the growth of *Y. enterocolitica* under PMB or PME at sub-inhibitory concentration.

**Table 2:** Minimal inhibitory concentration (MIC) and minimal bactericidal concentration (MBC) of wild type, *phoP* and *phoQ* mutants to PMB and PME.

	MIC (μg/ml)		MBC (μg/ml)	
	PMB	PME	PMB	PME
WT	2	2	16	16
Δ*phoP*	1	1	8	8
Δ*phoQ*	1	1	4	8

### Deletion of PhoP/PhoQ Increases Membrane Sensitivity and Decreases Intracellular Metabolism When Exposed to Polymyxin

**Figure 3:**
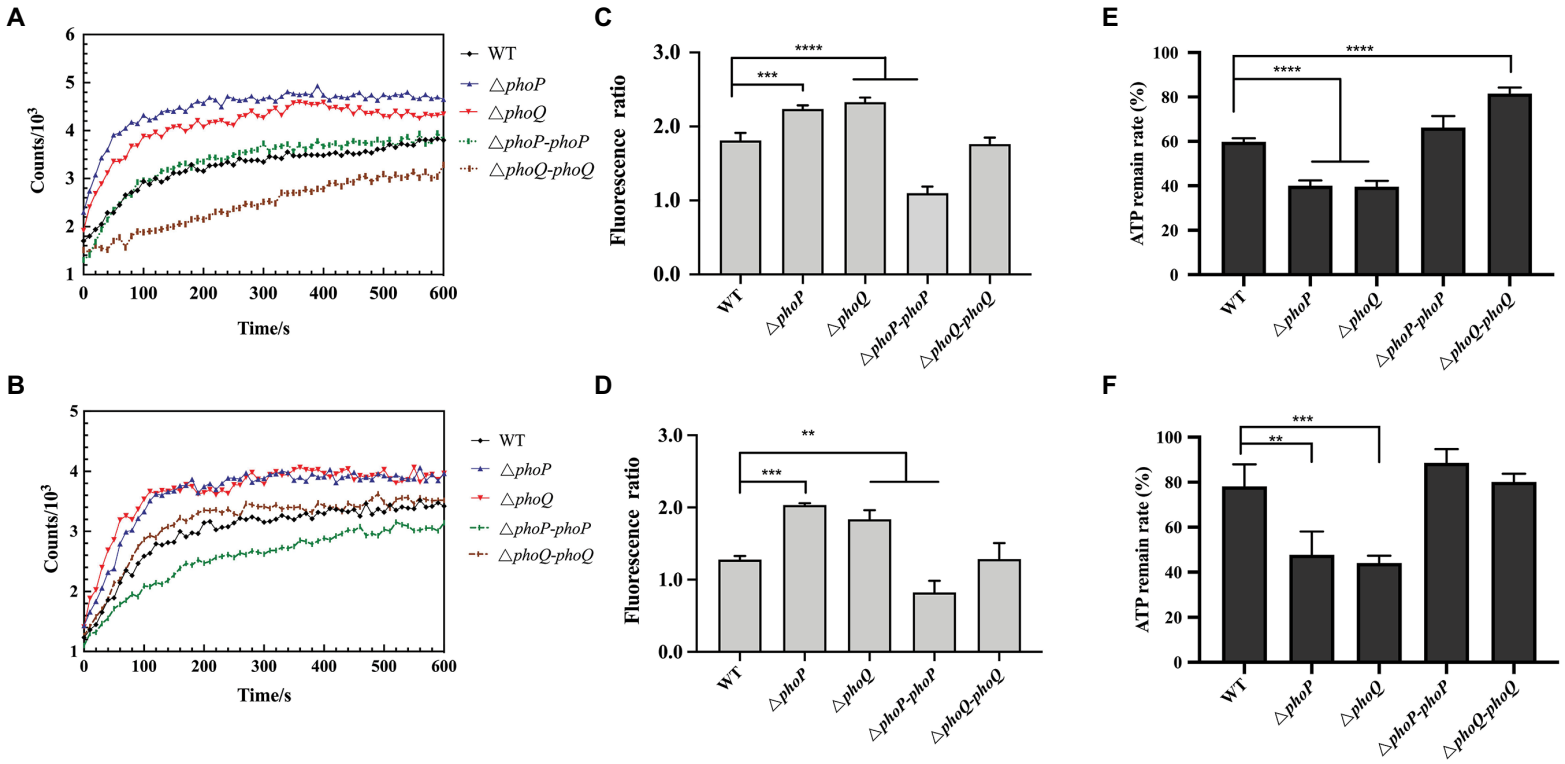
PhoP/PhoQ is essential for *Y. enterocolitica* to maintain membrane stability and intercellular metabolism when treated with 0.5 μg/ml PMB or PME. Outer membrane permeability evaluated by the N-phenyl-1-naphthylamine (NPN) uptake of wild type, mutants and complemented strains treated with 0.5 μg/ml PMB **(A)** and PME **(B)**. Membrane depolarization of wild type, mutants and complemented strains treated with 0.5 μg/ml PMB **(C)** and PME **(D)** and measured using DiBAC_4_(3). The ordinate is the fluorescence ratio between the treatment group and the blank (untreated with PMB or PME). The intercellular ATP remain rate following treatment with 0.5 μg/ml PMB **(E)** and PME **(F)**. Asterisks indicate a significant difference. ****, *p* < 0.0001; ***, *p* < 0.001; and **, *p* < 0.01.

According to several reports, polymyxin can cause membrane damage, thereby interfering with the normal physiological functions of bacteria (Santos et al., 2017). Therefore, the changes in cell membrane potential and cell membrane permeability of the wild-type and mutant strains under sub-inhibitory concentrations of PMB or PME were explored. When PME and PMB were stimulated with 0.5 μg/ml, the permeability of the outer membrane and the changes in the membrane potential were measured, the results of which are displayed in Figures 3A,B. The NPN uptake method was used to evaluate the permeability of the outer membrane. NPN is a hydrophobic fluorescent probe that can be embedded in the cell membrane (Lee and Je, 2013). As its fluorescence intensity increases in a hydrophobic environment, it is often used to reflect the permeability of the cell membrane (Lee and Je, 2013; Akhoundsadegh et al., 2019). The fluorescence of the *phoP* and *phoQ* mutants was significantly higher than that of the wild type. In addition, the absorbance at 410 nm of the mutants was higher than that of the wild type under PMB or PME (Supplementary Figure 3). Thus, PMB or PME can be concluded to cause severe damage to the membrane permeability of mutants compared to the wild type. The membrane potential dye, DiBAC_4_(3), was used to measure the cellular membrane potential of *Y. enterocolitica*. At 0.5 μg/ml of PMB or PME, the membrane depolarization of the mutants was significantly enhanced compared with that of the wild type (Figures 3C,D).

Treatment with AMP can reduce cytoplasmic ATP concentration and inhibit respiration (Brogden, 2005). Bacterial resistance to PMB has also been reported to be strongly related to energy metabolism (Yu et al., 2019). To further explore whether the PhoP/PhoQ system influences the intracellular metabolism of *Y. enterocolitica* under stimulation with the same sub-inhibitory concentration, the intracellular ATP contents of mutants and wild type were determined at 0.5 μg/ml PMB or PME, and the results are shown in Figures 3E,F. The intracellular ATP content of wild type and mutants were reduced after PMB or PME treatment, but the mutants showed a significantly higher degree of reduction when compared with the wild type. Therefore, the resistance of *Y. enterocolitica* to PMB or PME after the deletion of the PhoP/PhoQ system was observed, which was demonstrated by more severe changes in cell membrane permeability, membrane depolarization, and intracellular metabolism.

### PhoP/PhoQ Is Responsible for the Increased Resistance of Cell Membrane to Polymyxin Binding

**Figure 4:**
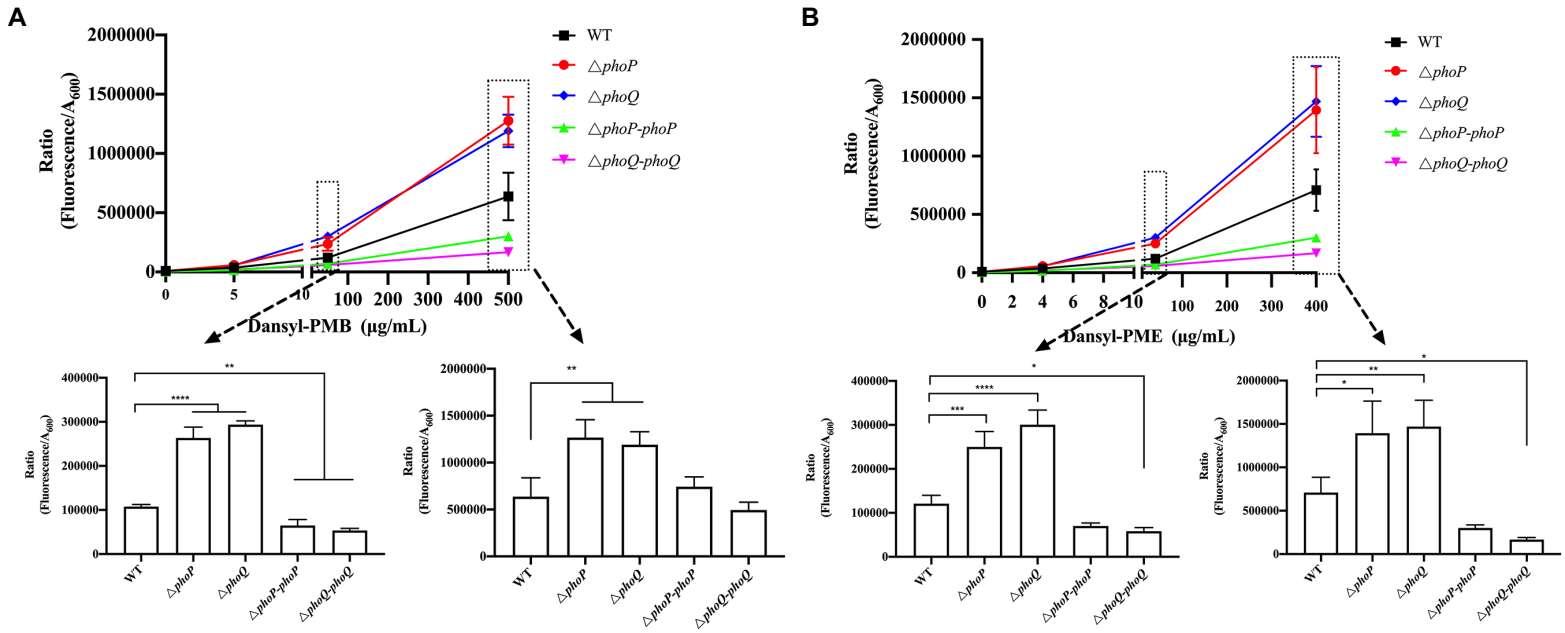
PhoP/PhoQ created a more resistant *Y. enterocolitica* membrane for the binding of polymyxin. The binding ability of different concentrations of Dansyl-PMB **(A)** and Dansyl-PME **(B)** to wild type, mutants and complemented strains. Asterisks indicate a significant difference. ****, *p* < 0.0001; ***, *p* < 0.001; **, *p* < 0.01; and *, *p* < 0.05.

Polymyxin binding to membrane promote cation displacement and structural changes in membrane curvature. And this binding is selectively which determines the sensitivity or resistance of bacteria to AMPs (Santos et al., 2017). The interactions between polymyxins and the Gram-negative outer membrane play an important role in polymyxin resistance (Brogden, 2005). Thus, the following hypothesis was proposed: the existence of the PhoP/PhoQ system can increase the resistance to polymyxins by reducing the binding and/or entry ability of polymyxin to *Y. enterocolitica*. To prove this hypothesis, PMB or PME binding assays were performed using the fluorescently labeled polymyxin compounds, Dansyl-PMB and Dansyl-PME. The quantification results of the binding or entry ability of Dansyl-PMB and Dansyl-PME with *Y. enterocolitica* are presented in Figure 4. First, the fluorescence intensity increased with increasing concentrations of Dansyl-PMB or Dansyl-PME, indicating increased surface-bound or entry of Dansyl-PMB or Dansyl-PME. No significant difference in fluorescence was found between the wild type and mutants when dansyl-PMB and dansyl-PME concentrations of 5 and 4 μg/ml were, respectively, employed. However, *phoP* and *phoQ* mutants showed significantly increased fluorescence compared to the wild type when the concentrations of dansyl-PMB and dansyl-PME were 50, 500, 40, and 400 μg/ml, respectively, confirming the protective effect of PhoP/PhoQ against polymyxin attack on the bacterial surface of *Y. enterocolitica*. Therefore, these results proved our hypothesis that the existence of PhoP/PhoQ provides more resistance to polymyxin binding in the cell membrane of *Y. enterocolitica*.

### LPS Structure, Which Is Associated With AMP Resistance, Depends on the Regulation of PhoP/PhoQ

**Figure 5:**
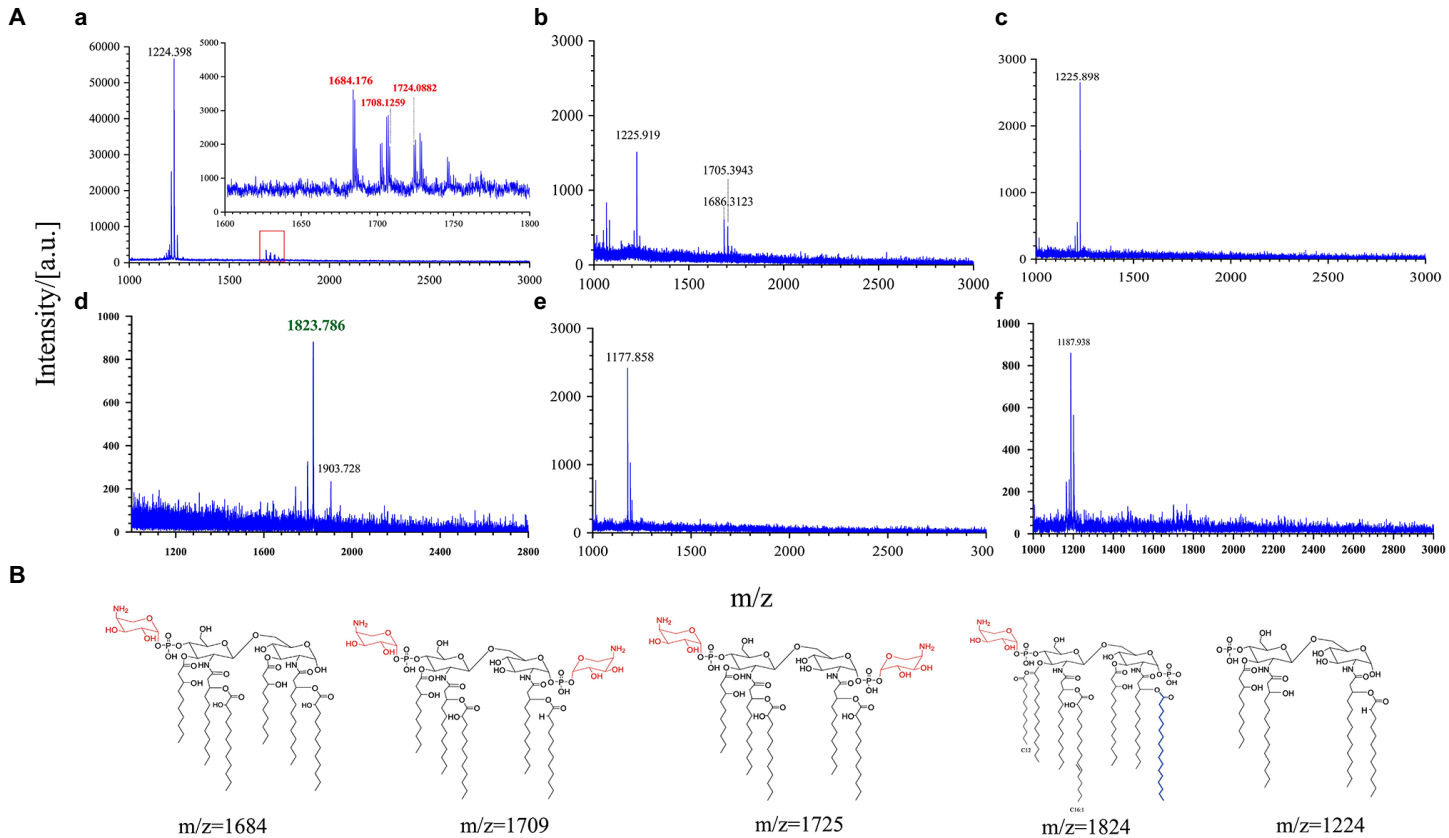
PhoP/PhoQ regulates *Y. enterocolitica* to form different lipopolysaccharide (LPS) modifications. MALDI-TOF analysis of wild type and mutants treated with PMB or PME **(A)**. **(a–c)** Display the results of wild type, *phoP* mutants, and *phoQ* mutants treated with PMB and **(d–f)** display the results of wild type, *phoP* mutants, and *phoQ* mutants treated with PME. Possible structures of lipid A at different *m*/*z* **(B)**.

The interaction of AMPs with the anionic bacterial surface is necessary for the exertion of their microbicidal action (Brogden, 2005). LPS modifications that reduce the negative charge or alter the acyl chains of lipid A provide resistance against AMP binding through charge repulsion or decrease the fluidity of the outer membrane (Simpson and Trent, 2019). To further explore the relationship between the binding ability of AMPs to the outer membrane of *Y. enterocolitica* and LPS modification, MALDI-TOF mass spectrometry was used to characterize the lipid A structure. Figure 5Aa shows that lipid A from *Y. enterocolitica* exposed to PMB contained a predominant peak at *m*/*z* 1,224. And three peaks were also found at *m*/*z* 1,864, *m*/*z* 1,708, and *m*/*z* 1,724 which contained two glucosamines, one or two phosphates, 2-OH-C12, and aminoarabinose (Figure 5B; Reinés et al., 2012a,b; Han et al., 2018a). The wild type grown under the PME showed a predominant peak at *m*/*z* 1,823 for a lipid A species that predominantly contained hexa-acylated species that correspond to two glucosamines, two phosphates, four 3-OH-C14, one C12, and one C16:1 (Figure 5B; Reinés et al., 2012b). The presence of polymyxins caused *Y. enterocolitica* to induce palmitate and aminoarabinose lipid A modification, which increased the resistance to polymyxins. Lipid A from wild type and mutants also did not undergo pEtN modification under PMB and PME stimulation, which may be related to the absence of PmrD in *Y. enterocolitica* (Reinés et al., 2012b). In the presence of PMB or PME, modifications to lipid A by aminoarabinose or palmitate could be detected in *Y. enterocolitica* under the regulation of PhoP/PhoQ, which led to resistance in polymyxin binding to the membrane and higher resistance. It was showed that lipid A from *phoP* and *phoQ* mutants under PME lacked multiple modifications, which could explain the higher fluorescence of the polymyxin binding mutants. Similarly, the *phoQ* mutants treated with PMB also lacked multiple modifications. However, for *phoP* mutants under PMB, result was also shown that peak at *m*/*z* 1,686 which was closely to *m*/*z* 1,684 has also been detected. This reveals that other pathways may involve in the regulation of lipid A modifications. Although PmrD is absent in *Y. enterocolitica*, but whether the cross-regulation between PhoP/PhoQ and PmrA/PmrB is existence has not been proved, so the quantification of the transcription level of PmrA/PmrB was performed which may give some explanation.

### Activated PhoP/PhoQ Upregulates Genes Related to LPS Modification in *Yersinia enterocolitica*

**Figure 6:**
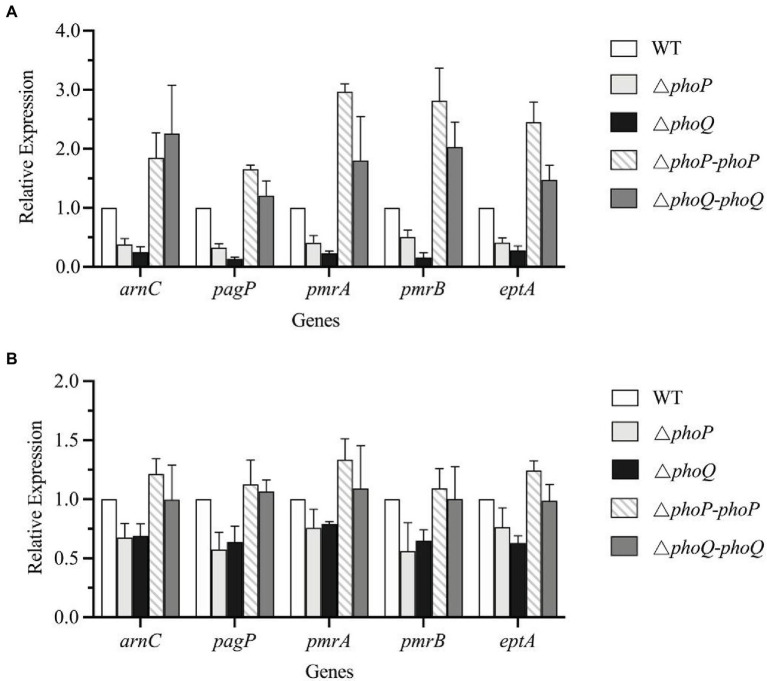
PhoP/PhoQ is involved in the regulation of genes related to LPS modification. Transcriptional changes of wild type, mutants and complemented strains treated with PMB **(A)** and PME **(B)**.

The above data confirmed that the presence of the PhoP/PhoQ system can increase the viability of *Y. enterocolitica* when exposed to PMB or PME. The increased resistance was achieved by reducing the binding ability of PMB or PME to *Y. enterocolitica*. The chemical characteristics of lipid A were characterized by MALDI-TOF, which revealed that wild-type *Y. enterocolitica* had a variety of structures related to AMP resistance compared to the mutants. Studies have shown that PhoP/PhoQ regulates *pagP* directly to maintain the asymmetry of the outer membrane (Simpson and Trent, 2019). Moreover, *arnBCADTEF* is required for the synthesis and addition of aminoarabinose to lipid A (Reinés et al., 2012a). *Salmonella enterica* subspp. and *E. coli* encode the protein, PmrD, which mediates coupling between PhoP/PhoQ and PmrA/PmrB (Rubin et al., 2015; Hong et al., 2018). Although PmrD was not found in *Y. enterocolitica* (Reinés et al., 2012b), the connections between PhoP/PhoQ and PmrA/PmrB can also be revealed using small RNAs, which remain unclear in *Y. enterocolitica*. In this study, the transcription levels of lipid A modification genes were analyzed to determine the regulatory effects of PhoP/PhoQ on lipid A modification (Figure 6). When exposed to PMB or PME at the MIC, the transcription levels of the genes, *pagP* and *arnC*, in the mutant strains were both reduced compared to those in the wild type. When exposed to PMB, *pagP* was downregulated by an average of 71 and 83% in the *phoP* and *phoQ* mutants, respectively, whereas *arnC* was downregulated by an average of 67 and 78%, respectively. The transcription levels of these genes in the Δ*phoP*-*phoP* and Δ*phoQ*-*phoQ* strains were restored. Exposed to PME, *arnC* was downregulated by an average of 32.4 and 30.8% in the *phoP* and *phoQ* mutants, respectively. And *pagP* was downregulated by an average of 42.5 and 36%, respectively, in *phoP* and *phoQ* mutants. There are differences of genes transcription between PMB and PME treatment. It may be caused by the differences of these two AMPs, as LPS modifications formed by *Y. enterocolitica* were also various under PMB and PME.

Since the absent of PmrD, we speculated that PmrA/PmrB and PhoP/PhoQ independently regulated LPS modification pathways and without interaction. But the transcription levels of *pmrA*, *pmrB*, and *eptA* in the mutants were reduced to varying degrees compared with the wild type, indicating that the deletion of PhoP/PhoQ in *Y. enterocolitica* can also affect the transcription of the PmrA/PmrB system.

## Discussion

Polymyxins have been employed as “last resort” antimicrobials (Han et al., 2018a). Gaining a better understanding of the mechanism of resistance to polymyxin is of critical importance. Currently, there are two main mechanisms to explain bacterial resistance to polymyxin: intrinsic resistance (adaptive resistance), which relies on LPS modifications, such as L-Ara4N and pEtN; and *mcr* genes induced resistance, which are encoded on plasmids and phages (Trimble et al., 2016; MacNair et al., 2018; Simpson and Trent, 2019). In this study, PhoP/PhoQ was demonstrated to be essential for the resistance of *Y. enterocolitica* to polymyxins (Figure 2). This was mainly because PhoP/PhoQ conferred a more resistant membrane for the binding of polymyxin by regulating LPS modification related to AMP resistance (Figures 4, 5). However, the LPS modification of *Y. enterocolitica* under PMB and PME was found to be different (Figure 5). In the presence of PMB, a variety of lipid-modified structures containing L-Ara4N were detected. However, when exposed to PME, in addition to containing L-Ara4N, the structures of palmitate and C16:1 were also observed (*m*/*z* at 1,823). Thus, when *Y. enterocolitica* was exposed to different AMPs, the LPS modification was not totally the same. And L-Ara4N was the main structure for *Y. enterocolitica* to set the adaptive resistance to polymyxin. Deletion of *phoP* and *phoQ* in *Y. enterocolitica* resulted the downregulation of the *arnBCADTEF* and *papP* which encode enzymes that responsible for L-Ara4N and palmitate (Figure 6). These results emphasized the role of PhoP/PhoQ in response to polymyxin in *Y. enterocolitica*. LPS modification also reduced the binding ability of polymyxin to *Y. enterocolitica* (Figure 4). This decrease may be due to a reduction in the negative charge and resistance to polymyxin induced by charge repulsion (Simpson and Trent, 2019). In addition, steric hindrance and lipid A tail packing produced by LPS modification could decrease the fluidity of the outer membrane, which may also prevent polymyxin from binding to *Y. enterocolitica* (Khondker et al., 2019; Simpson and Trent, 2019). Studies have shown that polymyxin exerts antimicrobial activity by targeting the bacterial cell membrane and gradually destroys the outer and inner membranes (Brogden, 2005). The destruction of the inner and outer membranes also increased when PhoP/PhoQ was absent (Figure 3; Supplementary Figure 2). Besides, study has reported that polymyxin B causes malfunction of respiration and severe consumption of ATP, leading to cell death and carbon starvation. And maintaining ATP level benefits the polymyxin resistance (Yu et al., 2019). Similarly, in Figures 3E,F, it is showed that the mutants of *phoP* and *phoQ* were sensitive to polymyxins and maintained lower intercellular ATP content compared to wild type.

**Figure 7:**
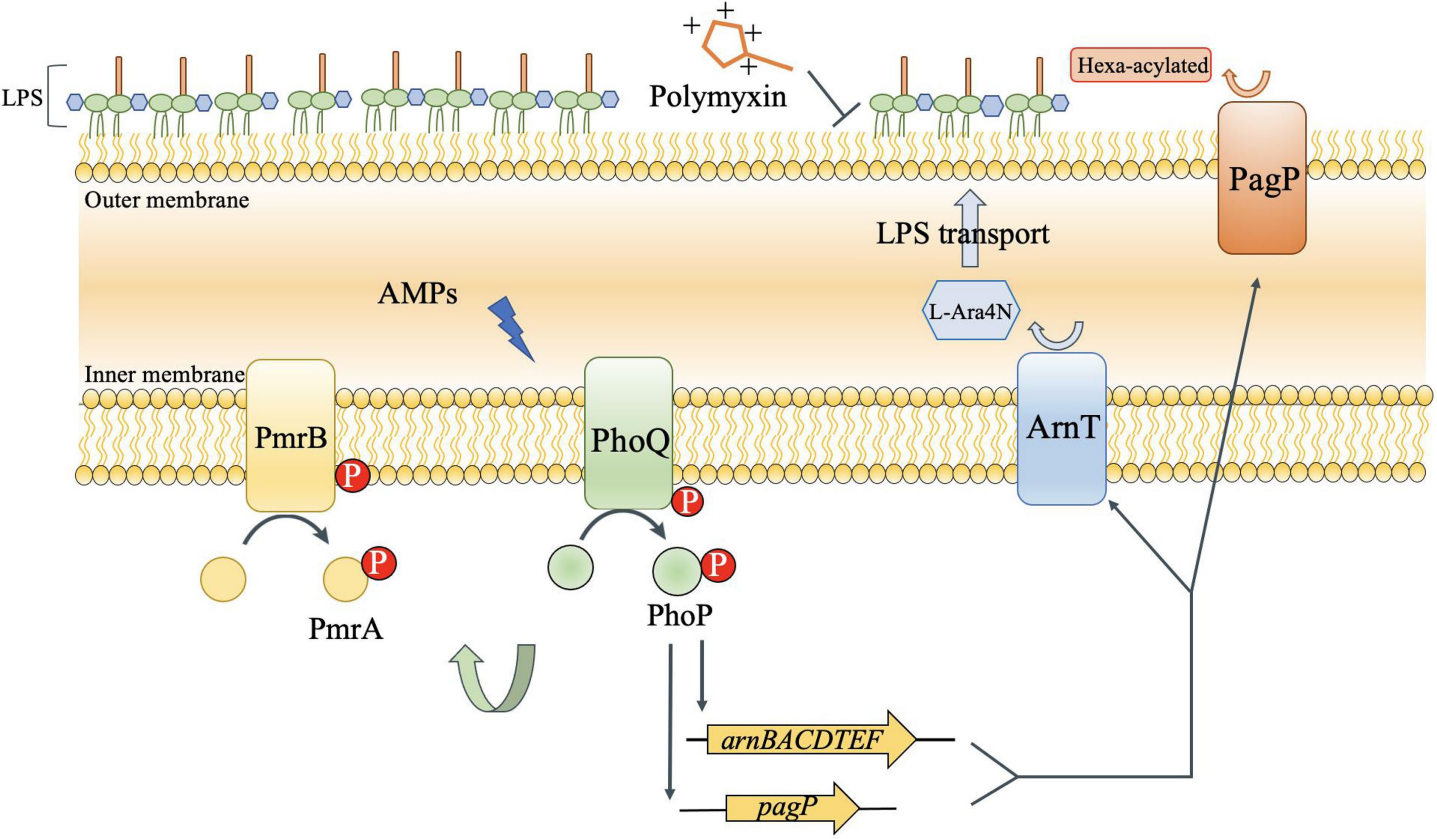
Regulation of LPS modification by the PhoP/PhoQ pathway of *Y. enterocolitica* to increase self-induced resistance to polymyxin. PhoP/PhoQ regulates genes that encode enzymes that alter acylation (*PagP*) and modify phosphates with aminoarabinose (*arnT*). The activity of PhoP can also affect the transcription of *pmrA* and *pmrB*. The increased modification of lipid A by *ArnT* and *PagP* alters the charge of the outer membrane and accumulates lipid tail packing to decrease the fluidity, which caused the resistance of *Y. enterocolitica* to AMP.

In most Gram-negative bacteria, the TCSs, PhoP/PhoQ and PmrA/PmrB, mediate polymyxin resistance by governing the LPS modification pathway (Trimble et al., 2016; Simpson and Trent, 2019). However, there are some differences in the LPS modification mechanisms among different Gram-negative bacteria, as shown in Figure 1. For example, PhoP phosphorylated in *Salmonella* can activate PmrA/PmrB through the protein, PmrD (Hong et al., 2018). Although PmrD in *E. coli* cannot function in the same manner as *Salmonella*, it is required for the modification of lipid A in *E. coli* under low Mg^2+^ growth conditions (Rubin et al., 2015). In *Klebsiella pneumoniae*, the *arn* operon can not only be regulated directly by PhoP/PhoQ, but also indirectly through PmrD (Chen et al., 2021). In *Yersinia pestis*, despite the lack of PmrD, protein lipid A with L-Ara4N can be promoted by PhoP dependently and independently through different inducing signals (Winfield et al., 2005). Based on our results, the role of PhoP/PhoQ in intrinsic resistance mechanism of *Y. enterocolitica* to polymyxins summarized as shown in Figure 7. The modification of LPS with L-Ara4N and palmitate regulated by PhoP/PhoQ increase the resistance of *Y. enterocolitica*. It reduces the binding ability of polymyxin to cell membrane. For the cross-regulation between PhoP/PhoQ and PmrA/PmrB, although PmrD was absent in *Y. enterocolitica*, and such pEtN structure was not observed under PMB and PME (Figure 5), the connection between PhoP/PhoQ and PmrA/PmrB was also observed. By comparing the results of gene expression analysis, the transcription of *pmrA*, *pmrB*, and *eptA* in the *phoP* and *phoQ* mutants was downregulated (Figure 6). Besides, for *Y. enterocolitica*, the intrinsic resistance mechanism induced by PhoP/PhoQ showed difference compared with *Salmonella*, not only the absent of PmrD, but also other genes such as *lpxT* lacking a PhoP binding site (Hong et al., 2018). And lipid A containing L-Ara4N has still been detected in *phoP* mutants (Figure 5), these all indicates that the response to polymyxin is not independently regulated by PhoP/PhoQ. Some non-coding small RNAs may play an important role in regulation pathways in *Y. enterocolitica* (Simpson and Trent, 2019). But, detail mechanism of the intrinsic resistance needs further study.

Although the regulation pathway of PhoP/PhoQ in the presence of polymyxin in *Y. enterocolitica* is not completely understood, we demonstrated the role of PhoP/PhoQ to increase the resistance of *Y. enterocolitica* to polymyxins. And we also raised some new tasks to find out the mechanism of the intrinsic resistance in *Y. enterocolitica*. Firstly, finding out the other potential regulatory factors involved in the connection between PhoP/PhoQ and PmrA/PmrB. Secondly, screening other regulatory factors involved in lipid A modifications which enhance the intrinsic resistance to polymyxins.

## Conclusion

All in all, it is important to gain a better understanding of the intrinsic resistance toward polymyxin in Gram-negative bacteria. In this study, the role of PhoP/PhoQ in *Y. enterocolitica* intrinsic resistance to polymyxins was demonstrated. The existence of PhoP/PhoQ in *Y. enterocolitica* showed a higher resistance to polymyxins, which was mainly caused by the modification of LPS with palmitate and L-Ara4N, and regulated by PhoP/PhoQ, conferring a more resistant membrane for the binding of polymyxin. According to the transcription analysis, the PhoP/PhoQ of *Y. enterocolitica* not only regulated LPS modification genes, but also affected the transcription of PmrA/PmrB. These findings expand our understanding of the intrinsic resistance to polymyxin in *Y. enterocolitica* and expand current knowledge regarding the role of PhoP/PhoQ in Enterobacteriaceae.

## Data Availability Statement

All datasets generated for this study are included in the article/Supplementary Material.

## Author Contributions

HG performed the experiments under the guidance of JC, analyzed the experimental data, and drafted the manuscript. JC and HG developed the idea for the study and designed the research. TZ and CH made substantial contributions to conception, interpretation of data and revised the manuscript. All authors have read and approved the final manuscript.

## Funding

This work was supported by the Beijing Natural Science Foundation (6202016) and the National Natural Science Foundation of China (31671830).

## Conflict of Interest

The authors declare that the research was conducted in the absence of any commercial or financial relationships that could be construed as a potential conflict of interest.

## Publisher’s Note

All claims expressed in this article are solely those of the authors and do not necessarily represent those of their affiliated organizations, or those of the publisher, the editors and the reviewers. Any product that may be evaluated in this article, or claim that may be made by its manufacturer, is not guaranteed or endorsed by the publisher.
