# Supplementary material for: The Role of the Two-Component System PhoP/PhoQ in Intrinsic Resistance of Yersinia enterocolitica to Polymyxin
Source: Front Microbiol. 2022 Feb 10;13:758571. doi: 10.3389/fmicb.2022.758571 (PMC8867023; doi:10.3389/fmicb.2022.758571)
Supplement: Supplementary file 1 [file Table_1.DOCX]

Supplementary Material


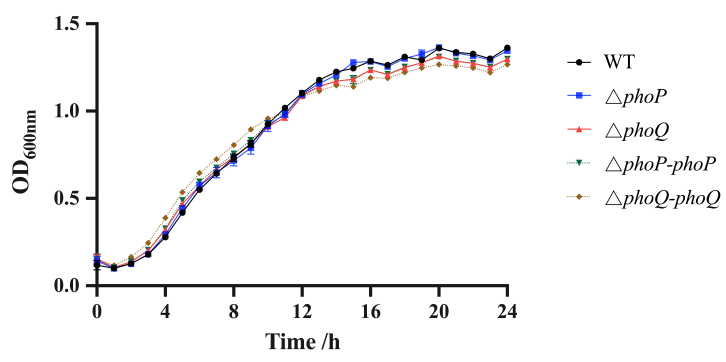


## Supplementary Figure 1 Growth curve of wild type, mutants, and complemented strains.

## Supplementary Figure 2 LCSM images of live and dead cells of *Y. enterocolitica* after staining with PI and DAPI after treatment with 5 μg/mL PMB or PME; images were captured at 1000× magnification (A). Quantification results of these images are presented on the right (B).

**(B)**

WT △*phoP*  △*phoQ*

**(A)**

WT △*phoP*  △*phoQ*

PMB

PME


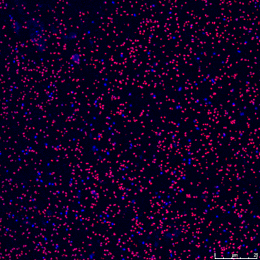

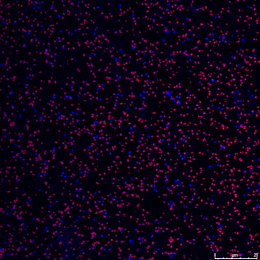

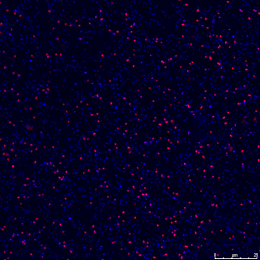

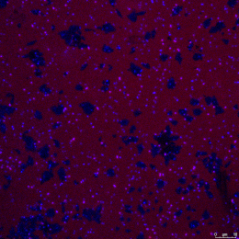

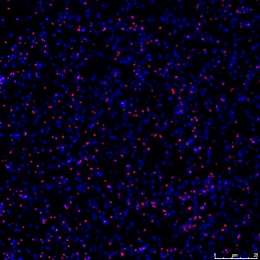

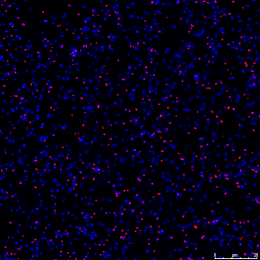

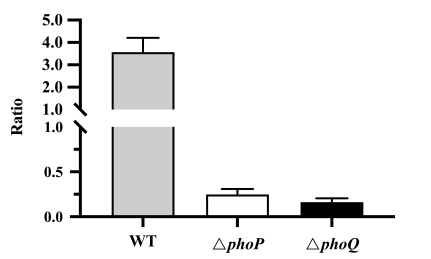

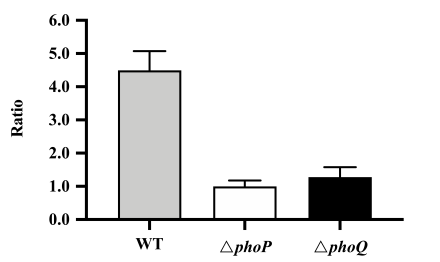


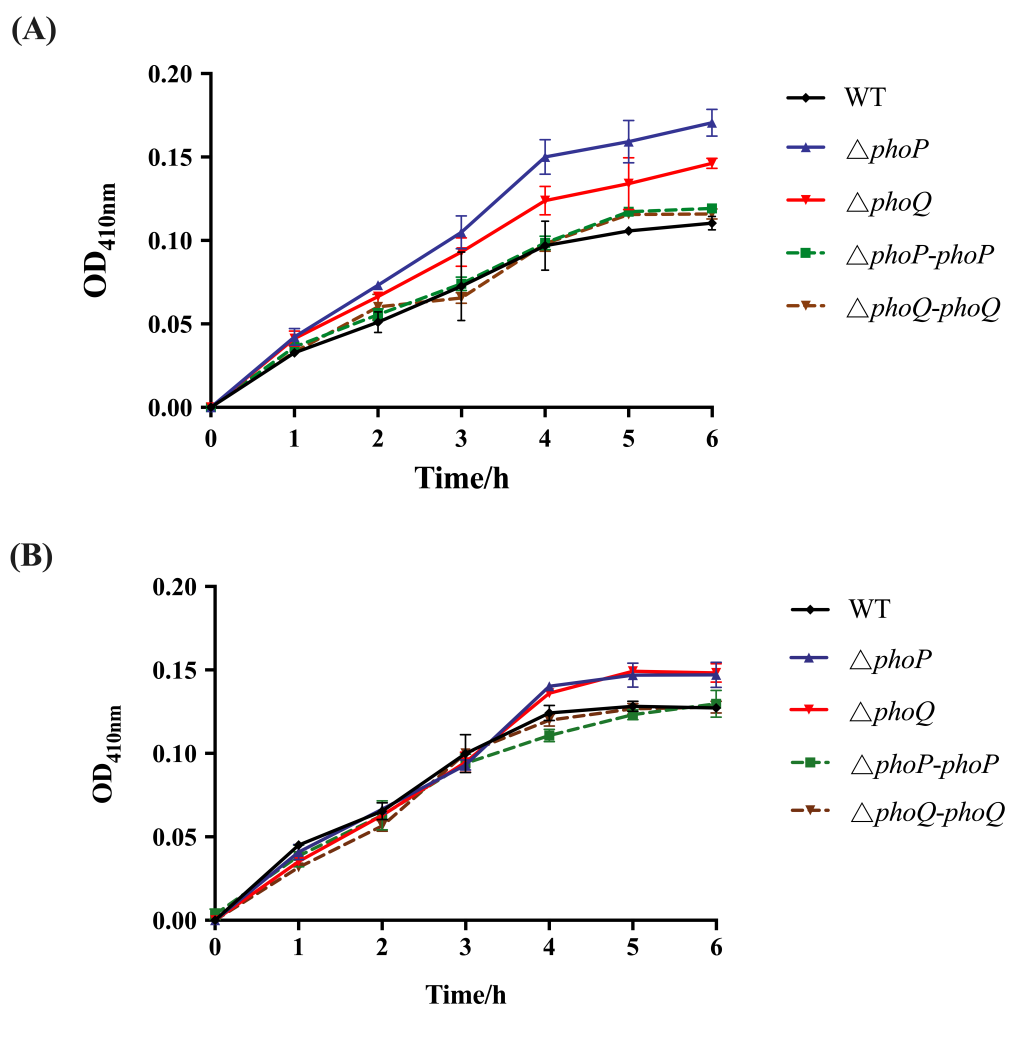
**Supplementary Figure 3** Inner membrane permeability evaluated by the release of β-galactosidase activity of the wild type and mutant strains under the treatment of 5μg/mL PMB(A) or PME(B).

**Supplementary Table 1** Primers used in this study

| **Primer** | **Sequence (5′→3′)** |
| --- | --- |
| *phoQ*-up-F | ATATAGAGCTCTTAAGCCTTATCCGTCGTTG |
| *phoQ*-up-R | GTAAATTTTAGATTTCTAGTTAGCGTCAAAAC |
| *phoQ*-down-F | ACTAGAAATCTAAAATTTACCTGCCAATAT |
| *phoQ*-down-R | GCGATGTCGACCGCCCCGTGCCCTGAATAAT |
| *phoP*-up-F | ATATAGAGCTCGGCTATGGTTTTGATAACGAC |
| *phoP*-up-R | AACATGATTTACTACATTCCTCGATAAGAC |
| *phoP*-down-F | GGAATGTAGTAAATCATGTTCAGGACAAAT |
| *phoP*-down-R | GCGATGTCGACGGATGTGGCGGGGTAAGTAT |
| *phoQ*-F | ATATATCTAGAATGTTCAGGACCAAATAGCAAG |
| *phoQ*-R | GCGATAAGCTTTTAATCAGCGTGATAATCAT |
| *phoP*-F | ATATATCTAGAATGCGGGTTTTAGTTGTCGAAG |
| *phoP*-R | GCGATAAGCTTCTAGTTAGCGTCAAAACGGT |

| **Primer** | **Sequence (5′→3′)** |
| --- | --- |
| *16S rRNA-F* | GCACGTAATGGTGGGAACTC |
| *16S rRNA-R* | CTCCAATCCGGACTACGACA |
| *q-pagP-F* | AGCCTATGCTTGGGTCAAGT |
| *q-pagP-R* | GCGTTGCCATAGGTTGTCAT |
| *q-arnC-F* | TCAATCAGGTCAGTGGCGAT |
| *q-arnC-R* | GATCATGCGGGAAGCTGTTT |
| *q-pmrA-F* | TCAAACCGTCAATCGTGAGC |
| *q-pmrA-R* | GGATGCGATCTTTGCCCAAT |
| *q-pmrB-F* | TGCCAGTGGCCTTTATCAGA |
| *q-pmrB-R* | GACGCTGAGCACACATTTCT |
| *q-eptA-F* | GGGAAACCAATCCACGACTG |
| *q-eptA-R* | CCGCGGCATATTTGAGAACA |

**Supplementary Table 2** RT-qPCR primers used in this study
